# Ferroptosis in breast cancer: From adipocyte–immune–iron regulation to therapeutic application

**DOI:** 10.1002/ctm2.70707

**Published:** 2026-06-01

**Authors:** Juan Sun, Yang Qu, Ru Yao, Yidong Zhou

**Affiliations:** ^1^ Department of Breast Surgery Peking Union Medical College Hospital Chinese Academy of Medical Sciences and Peking Union Medical College Dongcheng District Beijing China

**Keywords:** adipocytes, breast cancer, ferroptosis, immune cells, iron metabolism, precision medicine, tumour microenvironment

## Abstract

**Background:**

Ferroptosis, an iron‐dependent form of regulated cell death driven by lipid peroxidation, has emerged as a potential therapeutic vulnerability in breast cancer. However, increasing evidence indicates that ferroptosis sensitivity is not solely determined by tumour‐intrinsic factors, but is dynamically regulated by the tumour microenvironment (TME), particularly through interactions among adipocytes, immune cells and iron metabolism.

**Main body:**

Recent studies provide mechanistic evidence for this context dependence. Adipocyte‐derived monounsaturated fatty acids such as oleic acid suppress lipid peroxidation and increase resistance to ferroptosis induction in triple‐negative breast cancer, whereas ACSL4‐driven polyunsaturated phospholipid remodelling enhances ferroptosis susceptibility. In parallel, CD8^+^ T‐cell‐derived interferon‐γ promotes ferroptosis by suppressing SLC7A11‐mediated cystine uptake, while tumour‐associated macrophages buffer oxidative stress through iron sequestration and glutathione‐dependent antioxidant programs. These opposing forces indicate that ferroptosis is governed by a coordinated adipocyte–immune–iron regulatory network rather than a single pathway. Unlike previous reviews focused mainly on tumour–intrinsic mechanisms or general TME effects, this review integrates adipocyte‐derived lipid metabolism, immune‐mediated redox regulation, iron handling and spatial heterogeneity into a unified ‘ferroptosis ecosystem’ framework. Based on this concept, we propose eco‐ferrotherapy, a translational strategy aimed at simultaneously targeting tumour‐intrinsic pathways and microenvironmental buffering systems. This framework may support subtype‐specific therapeutic prioritisation, biomarker‐guided patient stratification and rational combination strategies involving immunotherapy and nanomedicine.

**Conclusion:**

Ferroptosis in breast cancer should be understood as an ecosystem‐level vulnerability shaped by metabolic, immune and spatial factors. Defining and therapeutically targeting this ferroptosis ecosystem provides a conceptual and translational roadmap for improving precision treatment strategies.

## Graphical abstract

Ferroptosis in breast cancer is regulated by an integrated adipocyte–immune–iron network rather than by tumour‐intrinsic pathways alone. Adipocyte‐derived lipid substrates and redox metabolites, immune‐cell‐mediated cytokine signalling and iron‐dependent oxidative stress collectively shape a dynamic ferroptosis ecosystem that can shift tumours towards either ferroptosis‐permissive or ferroptosis‐resistant states. Spatial heterogeneity and subtype‐specific metabolic programs further influence ferroptosis sensitivity. Based on this framework, eco‐ferrotherapy strategies integrating lipid targeting, immune modulation, iron regulation and nanomedicine‐based delivery may improve ferroptosis sensitisation and enhance precision therapy in breast cancer.

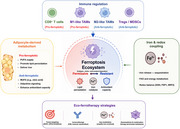

## 1 INTRODUCTION

Ferroptosis is an iron‐dependent form of regulated cell death driven by lethal lipid peroxidation and reactive oxygen species accumulation.^1^ Unlike apoptosis or necroptosis, ferroptosis is fundamentally governed by metabolic processes, in which iron homeostasis, lipid remodelling and antioxidant defence converge to determine cell fate.^2^ In breast cancer, this vulnerability is not solely dictated by tumour‐intrinsic programs but is strongly influenced by the surrounding tumour microenvironment (TME), which is characterised by abundant adipose tissue and dynamic immune infiltration.^3^ In particular, adipocyte‐derived fatty acids and immune‐cell‐mediated regulation of redox balance and iron handling can modulate the ferroptotic threshold of neighbouring tumour cells.^4^

Previous reviews have primarily focused on tumour‐intrinsic ferroptosis mechanisms, including lipid peroxidation pathways and glutathione peroxidase 4 (GPX4)‐dependent antioxidant systems, or have examined ferroptosis in the context of cancer immunity in a broader, cross‐tumour setting. While these studies have provided important mechanistic insights, they often consider individual regulatory layers in isolation and do not fully address how metabolic, immune and iron‐dependent processes are integrated within the breast cancer microenvironment. Moreover, the spatial organisation of adipose‐rich regions, immune‐cell‐enriched stromal niches and hypoxic tumour cores – an essential feature of breast cancer biology – has rarely been incorporated into current ferroptosis models.

To address this gap, we propose a unified adipocyte–immune–iron axis as a conceptual framework to explain how lipid metabolism, immune regulation and iron‐dependent oxidative processes collectively determine ferroptosis sensitivity in breast cancer. This framework is particularly relevant in an adipose‐rich tumour such as breast cancer, where subtype‐specific metabolic programs and spatial heterogeneity across adipose‐border regions, myeloid‐dominated stromal compartments and hypoxic cores may generate distinct ferroptosis‐permissive or ferroptosis‐resistant ecosystems. Emerging breast‐cancer studies support this view by demonstrating subtype‐dependent ferroptosis vulnerability, adipocyte‐mediated lipid buffering and macrophage‐associated redox and iron regulation.

In this review, we synthesise current evidence to (i) define how adipocyte‐derived lipid and redox support, immune‐cell‐mediated ferroptotic pressure or buffering and iron metabolism interact to regulate ferroptosis; (ii) highlight the context‐dependent and spatially organised nature of ferroptosis sensitivity; and (iii) discuss translational strategies, including eco‐ferrotherapy, biomarker‐guided patient stratification and rational combination therapies. To enhance transparency and address variability in evidence strength, we further introduce an evidence‐grading framework and provide five integrative tables summarising key mechanisms, context‐dependent effects, subtype‐specific strategies and a proposed clinical ferroptosis ecosystem score.

## 2 FERROPTOSIS IN BREAST CANCER: METABOLIC, MICROENVIRONMENTAL AND THERAPEUTIC LANDSCAPE

Ferroptosis is an iron‐dependent form of regulated cell death driven by lipid peroxidation and redox imbalance, representing a metabolic vulnerability in cancer cells.^1^, ^2^ A curated summary of breast‐cancer‐related evidence, annotated by experimental context and validation level, is provided in Table 1. In breast cancer, ferroptosis sensitivity is primarily governed by three interconnected processes: iron availability, lipid composition and antioxidant capacity. Dysregulated iron metabolism expands the labile iron pool and promotes Fenton chemistry, thereby enhancing oxidative stress; for example, iron‐delivery systems and catalytic nanoplatforms can amplify ROS generation and trigger ferroptosis in triple‐negative breast cancer (TNBC) models,^5^, ^6^ whereas selenium‐dependent GPX4 activity counteracts lipid peroxidation and stabilises cellular redox balance.^7^ In parallel, lipid remodelling defines substrate availability, with ACSL4/LPCAT3‐driven incorporation of polyunsaturated fatty acids (PUFAs) promoting peroxidation, while monounsaturated lipids derived from adipocytes confer resistance through membrane remodelling.^8^, ^9^, ^10^ Nutrient stress further modulates this balance by limiting cystine uptake and glutathione (GSH) synthesis, thereby lowering the threshold for ferroptotic induction.^11^, ^12^

**TABLE 1:** Representative studies of ferroptosis in breast cancer.

Year	Model/context	Key mechanism	Major molecules	Effect on ferroptosis	Model/validation level
Molecular and metabolic regulation					
2022^10^	Co‐culture of mammary adipocytes and TNBC cells	Adipocyte‐derived oleic acid inhibits lipid peroxidation via ACSL3‐dependent lipid remodelling	Oleic acid, ACSL3, lipid ROS	Suppresses	In vitro
2022^47^	TNBC cells and TAM co‐culture	TGF‐β1/HLF–GGT1–GPX4 axis induces antioxidant defence; IL‐6–JAK2/STAT3 maintains loop	TGF‐β1, HLF, GGT1, GPX4	Suppresses	In vitro + in vivo
2023^13^	TNBC subtype analysis (multi‐omics + PDOs)	AR–GPX4 axis suppresses ferroptosis in LAR subtype; GPX4 inhibition restores ferroptosis and enhances PD‐1 response	AR, GPX4, GSH	Suppresses in AR‐high	Multi‐omics + PDO + preclinical
2023^14^	HER2^+^ breast cancer resistance models	Integrin αvβ3 mediates AKT activation and ferroptosis resistance; inhibition restores sensitivity	ITGB3, AKT, SLC3A2, GSH, GPX4	Suppresses	In vitro + in vivo
2024^97^	BC fibroblast–tumour crosstalk	TDO2^+^ fibroblasts secrete KYN to activate AHR–FTH1 axis, conferring ferroptosis resistance	TDO2, KYN, AHR, FTH1, GPX4	Suppresses	In vitro + in vivo
2024^11^	Acidosis‐induced ferroptosis in BC	ZFAND5‐mediated SLC3A2 degradation decreases cystine import and GSH synthesis	ZFAND5, SLC3A2, GSH, lipid ROS	Promotes	In vitro + in vivo
2023^12^	FKBP1A/SLC3A2–everolimus axis in BC	Everolimus binds FKBP1A, downregulates SLC3A2 and GPX4, increases ROS and ferroptosis	FKBP1A, SLC3A2, GPX4, ROS	Promotes	In vitro
2024^6^	Copper–peroxide silica nanoparticles (DOT@DOX)	DOT releases Cu^2^^+^ and H_2_O_2_ for Fenton reaction; DOX induces apoptosis and enhances ferroptosis	Cu^2^^+^, H_2_O_2_, GSH, GPX4	Promotes	Preclinical nanotherapy
2025^15^	HR^+^HER2^−^ PF‐resistant BC cells	GPX4 upregulation drives palbociclib–fulvestrant resistance; ferroptosis inducers resensitise tumours	GPX4, FABP6, PPARγ, ROS	Suppresses	In vitro + translational preclinical
2025^18^	TNBC exosomes–macrophage crosstalk	Exosomal FOXM1 induces IDO1→KYN→NRF2 activation, inhibits ferroptosis and promotes M2 polarisation	FOXM1, IDO1, KYN, NRF2, GPX4	Suppresses	In vitro + in vivo
2025^49^	Chemoresistant BC models	IL1β^+^CD4^+^ T cells induce neutrophil ferroptosis via the IL1β/IL1R1/NF‐κB–MBOAT1 axis, promoting immunosuppression and chemoresistance	IL1β, NF‐κB, MBOAT1, PGE2	Context‐dependent	In vivo
2023^17^	TNBC cells/4T1 + anti‐PD‐1	PRMT5 methylates KEAP1 and activates NRF2 signalling, increasing GPX4‐associated antioxidant buffering	PRMT5, KEAP1, NRF2, GPX4	Suppresses	In vitro + in vivo
2021^5^	MDA‐MB‐231/xenograft	Holo‐lactoferrin increases iron uptake & LPO; Apo‐Lf antioxidant	Holo‐Lf, TfR, ferritin, ROS, MDA	Promotes (holo‐Lf); suppresses (apo‐Lf)	In vitro + in vivo
2025^57^	TNBC + TME macrophages	HEBP2–FOXA1–GSTP1 supports tumour ferroptosis resistance, glutamine competition promotes CCL3^+^ macrophage ferroptosis	HEBP2, FOXA1, GSTP1, CCL3	Context‐dependent	In vivo
2025^98^	BC bone metastasis model	PRODH2‐mediated metabolism promotes YY1 acetylation and increases SLC7A11 expression	PRODH2, YY1(K230ac), SLC7A11	Suppresses; PRODH2 blockade promotes ferroptosis	In vivo
2024^99^	Mutant p53 mice/cells	Mut‐p53 + NRF2 co‐activates Mgst3/Prdx6 antioxidant axis	p53(mut), NRF2, Mgst3, Prdx6	Suppresses	In vivo
2024^19^	ORI + RSL3 in BC cells	ORI activates JNK and suppresses NRF2/HO‐1→sensitises RSL3	ORI, RSL3, JNK, NRF2, HO‐1	Promotes	In vitro
2023^21^	Multi‐cohort + TNBC cells	MTHFD2 maintains NADPH/GSH and GPX4/SLC7A11	MTHFD2, SLC7A11, GPX4, NRF2	Suppresses; knockdown promotes	BC cohort + in vitro
2025^20^	TNBC cells/mice	RAB10 maintains ferroptosis resistance through the Slc37a2/mTOR axis and support of GPX4/HMGCR homeostasis	RAB10, Slc37a2, mTOR, GPX4, HMGCR	Suppresses; depletion promotes	In vitro + in vivo
2025^100^	TNBC cells	Oxidative stress‐induced ZEB1 acetylation increases NADPH/GSH buffering and decreases ACSL4	ZEB1, CBP, SIRT1, ACSL4, GPX4	Suppresses	In vitro + in vivo
2019^101^	HER2^+^ model	Neratinib induces ACSL4‐dependent ferroptosis and inhibits metastasis	Neratinib, ACSL4, iron markers	Promotes	In vivo
2025^102^	TNBC cells	ASCL1→p‐CREB1→GPX4 ↑; ASCL1 knockdown sensitises to paclitaxel	ASCL1, CREB1(pS133), GPX4	Suppresses; ASCL1 inhibition promotes	In vitro
Immune crosstalk within tumour microenvironment					
2021^22^	eNVs‐FAP immunotherapy in 4T1 tumours	IFN‐γ from CTLs downregulates SLC7A11/SLC3A2/GPX4, inducing ferroptosis and reducing CAF‐mediated resistance	IFN‐γ, SLC7A11, SLC3A2, GPX4	Promotes ferroptosis	In vivo
2023^48^	Acod1–itaconate pathway in tumour‐infiltrating neutrophils	GM‐CSF–STAT5–C/EBPβ→Acod1–itaconate→Nrf2/GPX4‐mediated ferroptosis resistance	Acod1, itaconate, Nrf2, GPX4	Suppresses in TINs	In vivo/mechanistic
2024^23^	Complement C5a/C5aR pathway	C5a–C5aR activation induces NRF2–GPX4 upregulation, promoting ferroptosis resistance and M2 polarisation	C5a, C5aR, NRF2, GPX4	Suppresses	In vivo
2025^24^	CAF‐exosomes→TNBC cells	CAF‐derived exosomal miR‐454‐3p targets ACSL4 and increases FSP1/GPX4‐associated ferroptosis resistance	exo‐miR‐454‐3p, ACSL4, FSP1, GPX4	Suppresses	In vitro/single‐study evidence
Nanomaterial‐mediated ferroptosis therapy					
2024^26^	4T1, RAW264.7, BMDCs; BALB/c (REV@SR780Fe@LEV‐RS17)	pH‐activatable PDT + Fe^3^^+^ release; GSH depletion→GPX4↓; ICD + cGAS–STING enhances immunity	SR780Fe, REV, Fe^3^^+^/Fe^2^^+^, GPX4, LPO, IFN‐β	Promotes	BC preclinical nanotherapy
2025^25^	TME remodelling via MPPC@CM nanozyme	Pt/Pd catalysis depletes GSH, promotes ROS; ICD and DC maturation amplify ferroptosis	GSH, GPX4, ROS, IFN‐γ	Promotes	BC preclinical nanotherapy
2025^103^	4T1 & HUVEC/HepG2; BALB/c (T‐T@Cu)	TA/TCNQ/Cu^2^^+^ nanocomplex; Cys/GSH‐responsive Cu^2^^+^ burst; NIR‐II mild PTT boosts ROS/LPO; ATP7A/7B↓→Cu efflux↓	TA, TCNQ, Cu^2^^+^, GPX4, DLAT agg., FDX1/LIAS, MDA	Promotes	BC preclinical nanotherapy
2025^104^	4T1/MDA‐MB‐231 (DLN)	Natural naphthoquinone induces iron overload + system Xc^−^/GPX4 suppression	FTH1 (iron load), SLC3A2, GPX4, ROS/LipROS	Promotes	BC preclinical
2025^105^	4T1/HUVEC; BALB/c (AsV nanodots)	As^3^^+^ depletes Cys/GSH; V^4^^+^/V^5^^+^ Fenton‐like ·OH; ICD‐linked	As^3^^+^, V redox, GSH, GPX4, MDA, DAMPs	Promotes	BC preclinical nanotherapy
2025^106^	MDA‐MB‐231 (AKR1B10 axis)	AKR1B10→AKT/GSK3β→NRF2→GPX4↑; OSU‐T315 reverses	AKR1B10, NRF2/GPX4; OSU‐T315→GPX4	Suppresses; OSU‐T315 promotes	In vitro
2025^63^	4T1–TAM microfluidic (SECM)	M2‐TAMs restore SLC7A11/GSH, clear ROS, protect membrane	SLC7A11, GSH efflux, ROS; static blocks	Suppresses; STAT3 inhibition promotes	Microfluidic/in vitro
2023^107^	FeOOH/siPROM2@HA targets BCSCs	Promotes iron uptake, blocks export, depletes GSH	FeOOH, siPROM2, HA‐CD44	Promotes ferroptosis	BC preclinical nanotherapy
Multimodal synergistic therapies (PTT/PDT/SDT/RT/chemo)					
2024^108^	CFA‐MN microneedle system	Laser‐triggered ROS and Fe^2^^+^ release induce synergistic apoptosis–ferroptosis via Fenton reaction and GSH depletion	Fe^2^^+^, OH, ^1^O_2_, GPX4, ACSL4	Promotes (PTT/PDT)	BC preclinical
2024^109^	CuO_2_@G5‐BS/TF + MR	CA‐IX targeting; self‐H_2_O_2_; Fe^3^^+^/Cu^2^^+^ CDT + PTT	Cu^2^^+^/Fe^3^^+^, LPO, GPX4	Promotes (CDT/PTT)	BC preclinical
2025^110^	DMFD@MN + DOX + PTT	Fe^2^^+^ Fenton + thermal; MDR reversal (P‐gp/HSP70↓)	Fe^2^^+^/OH, GPX4, GSH	Promotes (chemo/PTT)	BC preclinical
2024^111^	PTFTH + mild PTT	HA/CD44 targeting; Fe‐TA‐driven Fenton chemistry plus TRIM37‐siRNA	Fe^2^^+^/OH, GPX4, GSH	Promotes (CDT/PTT)	BC preclinical
2024^112^	CPIR NTG + NIR	CaO_2_/O_2_/NO supply plus RSL3 and photothermal enhancement	O_2_/NO, GPX4, LPO	Promotes (PTT/NO/PDT)	BC preclinical
2025^113^	MnO_x_‐Hy NR + 660 nm	GSH scavenging plus O_2_ supply boosts PDT‐driven lipid peroxidation	GSH, GPX4 inactivation, LPO	Promotes (PDT)	BC preclinical
2023^114^	FeP@Pt@HA + PTT/RT	Fe Fenton chemistry plus Pt catalase‐like activity enhances RT sensitisation	Fe^3^^+^/Fe^2^^+^, O_2_, LPO	Promotes (PTT/RT)	BC preclinical
2024^115^	Zn‐A4@FRT + 660 nm	ZPP‐PDT plus Zn^2^^+^‐CDT plus BNM chemotherapy	Zn^2^^+^/·OH, GPX4, MDA	Promotes (PDT/CDT/chemo)	BC preclinical
2024^116^	BSNPs + 660 nm + RT	Disulfide‐mediated GSH depletion plus SLC7A11 suppression and singlet oxygen	SLC7A11, GSH, GPX4, ^1^O_2_	Promotes (PDT/RT)	BC preclinical
2024^117^	Lipo‐MT‐SNAP + light	NO + •O_2_^−^→ONOO^−^; ICD promotes antitumour immunity	ONOO^−^, GPX4, GSH	Promotes (PDT/RNS)	BC preclinical
2025^118^	LDH@Au + SDT	Cascade nanozyme induces Ca^2^^+^ overload, oxidative stress and ferroptosis	Fe^2^^+^/·OH, GPX4, MDA	Promotes (SDT)	BC preclinical
2024^119^	CR‐736‐Fe^3^^+^@PEG@CREKA + PTT	Lysosome‐localised heat + Fe‐Fenton; HSP block	Fe^3^^+^/Fe^2^^+^, GPX4, LPO, HSP	Promotes (PTT/CDT)	BC preclinical
2024^120^	^TK^NP_DHA_‐Fc	Lysosome‐localised heat plus Fe‐Fenton chemistry and HSP inhibition	Fe^2^^+^/·OH, GPX4, GSH	Promotes (chemo‐loop)	BC preclinical
2024^121^	IR780/Ce@EGCG/APT	Mito‐ROS + HSP/GPX4 suppression with Ce redox	IR780, Ce redox, EGCG, GPX4	Promotes (PTT/PDT)	BC preclinical
2022^122^	PFTT@CM (PDT + TPZ)	Fe^3^^+^ Fenton + PDT + chemo synergy	Fe‐TCPP, TPZ, ROS	Promotes	BC preclinical
2023^123^	ExoCAR/T7@Micelle	BBB penetration; PDT (ROS) triggers RSL3 release	Ce6, RSL3, ExoCAR, T7	Promotes	BC metastasis preclinical
2025^124^	Bi_2_Se_3_ + RSL3 + RT	High‐Z RT sensitisation + GPX4 block→STING activation	Bi_2_Se_3_, RSL3, STING	Promotes	BC preclinical
2025^125^	EXO@CAT (SDT + ACSL4)	^1^O_2_ generation plus ACSL4‐mediated lipid donor effect amplifies lipid peroxidation	TCPP, ACSL4, CAT	Promotes	BC preclinical
2025^126^	Fe‐PDA‐MET + PTT	Fe^3^^+^→Fe^2^^+^ Fenton + thermal synergy; MET remodels TIME	Fe‐PDA, MET	Promotes	BC preclinical
2025^127^	ssP‐tHB@Fe/DOX	pH/GSH‐responsive Fe^3^^+^ reduction + DOX‐NOX4→H_2_O_2_↑	Fe^3^^+^/Fe^2^^+^, DOX, tHB	Promotes	BC preclinical
2021^128^	HMPB@Lip + NIR	Fenton‐independent lipid peroxidation catalysis + PTT	HMPB (Fe^2^^+^/Fe^3^^+^)	Promotes	BC preclinical
2024^129^	MSNs@Fe^2^^+^@DOX	CDT (Fenton) + chemo co‐delivery synergy	Fe^2^^+^, DOX, MSN	Promotes	BC preclinical
2024^130^	HfO_2_@MnO_2_@Gox + RT	MnO_2_ GSH depletion + GOx H_2_O_2_ supply + RT trigger	HfO_2_, MnO_2_, GOx	Promotes	BC preclinical
2025^131^	PTX@CPG (PDT + chemo)	Ce6‐ROS + paclitaxel GSH depletion→suppress GPX4	Ce6, PTX	Promotes	BC preclinical
2025^132^	PDA@Cu + RT	Cu‐mediated cuproptosis + ROS/GSH depletion	Cu^2^^+^/Cu^+^, FDX1, DLAT	Promotes	BC preclinical
Integration with immunotherapy					
2025^89^	LP‐CaP@iBEFT + anti‐PD‐L1	Triple‐pathway ferroptosis (GPX4/FSP1/DHODH) + Fe^3^^+^ Fenton; ICD elevates DC/CD8^+^; PD‐L1 blockade augments killing	GPX4, FSP1, DHODH, Fe^2^^+^/·OH, MDA; CD8^+^, IFN‐γ/TNF‐α	Promotes	BC preclinical immunotherapy
2024^133^	HM/Ef/LNT‐MOF‐MIL‐101(Fe) + anti‐PD‐1	Fe ion release + IFN‐γ from LNT‐activated T cells; enhances ICB	Fe^2^^+^/Fe^3^^+^, IFN‐γ, GSH, GPX4, MDA	Promotes	BC preclinical immunotherapy
2025^134^	HV NPs + αPD‐1	Hemin‐boosted PDT induces ICD; αPD‐1 reduces Tregs and sustains CD8^+^	GSH, GPX4, LPO; CRT/HMGB1/ATP; CD8^+^, Treg	Promotes	BC preclinical immunotherapy
2024^135^	GOx‐IA@HMON@IO + αPD‐L1	GSH‐responsive Fe cascade + glucose starvation (ferroptosis‐linked disulfidptosis)	Fe^2^^+^/·OH, GSH, GPX4; IFN‐γ→SLC7A11	Promotes	BC preclinical immunotherapy
2024^136^	CCLT@FT + US + αPD‐L1	Co‐redox ferroptosis + SDT ROS; lactate↓ neutralises TME; ICB elevates IFN‐γ	Co^2^^+^/Co^3^^+^, GSH, GPX4, LPO; IFN‐γ	Promotes	BC preclinical immunotherapy
2025^95^	IFNγ–CAMK2–PSAT1 axis	PSAT1 hydroxylation (P159‐OH) stabilises GPX4→resists IFNγ‐induced ferroptosis; CAMK2 blockade restores	CAMK2, PSAT1(pS337), GPX4(P159‐OH)	Suppresses; CAMK2 blockade promotes	BC mechanistic + immunotherapy preclinical
2025^94^	FASN–USP5 pathway	FASN palmitoylates USP5→de‐ubiquitinates GPX4→stabilisation; Orlistat reverses	FASN, USP5(palm.), GPX4	Suppresses; FASN/USP5 blockade promotes	BC mechanistic + immunotherapy preclinical
2025^137^	Cu‐nanozyme + OME + αPD‐1	Cu redox enzymatic cascade→ferroptosis/cuproptosis + ICD → immune boost	Cu‐nanozyme, OME, αPD‐1	Promotes	BC preclinical immunotherapy
Clinical/omics‐based ferroptosis profiling					
2025^16^	HR^+^ BC multi‐cohort	LMF_index stratifies immune & prognostic subtypes	7‐gene panel (KRT5, KLRB1 etc.); ACSL4	Low LMF_index→ACSL4↑→↑ ferroptosis sensitivity	BC multi‐cohort
2022^138^	BC vs BCBM cohorts	Ferroptosis‐related 14‐gene score linked to immune infiltration & drug sensitivity	LPCAT3, TFRC, HMOX1 …	High score→↑ immune infiltration and distinct drug profiles	BC cohort analysis
2022^139^	TNBC CDKN2A subtypes	“Cold / IFNγ / FTL‐dominant” molecular clusters	CDKN2A, FTL …	FTL‐dominant→↑ ferroptosis activity	BC cohort analysis
2022^140^	TCGA + CPTAC pan‐cancer analysis	CSPP1 overexpression suppresses ferroptosis and correlates with immune‐suppressive TME	CSPP1, GPX4, FTH1, SLC7A11	Suppresses	Mixed/not breast‐cancer‐specific only
2024^141^	PPAR signalling in TNBC (TCGA–GDSC)	PPAR activation enhances lipid metabolism and ferroptosis‐related gene expression	PPAR α/δ, RXR, ACSL1/3, CPT1A	Context‐dependent	BC bioinformatic analysis

Abbreviations: BC, breast cancer; TNBC, triple‐negative breast cancer; CAF, cancer‐associated fibroblast; PDO, patient‐derived organoid; ICD, immunogenic cell death; PTT, photothermal therapy; PDT, photodynamic therapy; SDT, sonodynamic therapy; RT, radiotherapy; TAM, tumour‐associated macrophage; TIME, tumour immune microenvironment.

Ferroptosis vulnerability is not uniform across breast cancer subtypes but instead reflects lineage‐specific metabolic programs and therapy‐induced adaptations. In TNBC, ferroptosis phenotypes are heterogeneous rather than uniformly elevated; multi‐omics analyses have shown that the luminal androgen receptor (LAR) subtype combines increased oxidised phospholipids with enhanced GSH metabolism and GPX4 dependence, supporting subtype‐directed ferroptosis sensitisation strategies.^13^ In HER2‐positive disease, targeted therapies can induce ferroptosis but may also trigger adaptive resistance through integrin αvβ3–AKT signalling and reprogramming of iron and antioxidant pathways.^14^ In HR^+^/HER2^−^ tumours, ferroptosis is more closely linked to endocrine and CDK4/6 inhibitor resistance, where increased GPX4 dependence creates a context for therapeutic re‐sensitisation, and lipid metabolism‐associated signatures may help identify tumours with greater immune activity and potential responsiveness to immunotherapy.^15^, ^16^ These observations indicate that ferroptosis is better understood as a dynamic and treatment‐dependent vulnerability rather than a fixed intrinsic property.

At the molecular level, ferroptosis thresholds are further tuned by antioxidant signalling and microenvironmental inputs. Breast cancer cells rely on SLC7A11–GSH–GPX4 and NRF2‐centred networks to buffer oxidative stress. For instance, PRMT5‐mediated KEAP1 methylation stabilises NRF2 and enhances GPX4 expression, while macrophage‐derived signals can reinforce GSH metabolism through FOXM1–IDO1–KYN–NRF2 signalling.^17^, ^18^ Conversely, disruption of these pathways restores ferroptotic sensitivity by suppressing NRF2/GPX4 activity.^19^, ^20^, ^21^ Immune and stromal cues impose an additional regulatory layer: interferon‐γ (IFN‐γ) released by CD8^+^ T cells promote ferroptosis through repression of SLC7A11, whereas myeloid‐cell pathways such as Acod1–itaconate and complement signalling enhance antioxidant defences and limit ferroptotic stress.^22^, ^23^ Stromal interactions also contribute, as CAF‐derived exosomal signals can suppress lipid peroxidation, while immunogenic nanoplatforms amplify ferroptosis through coupled activation of innate immune pathways.^24^, ^25^, ^26^

**FIGURE 1:**
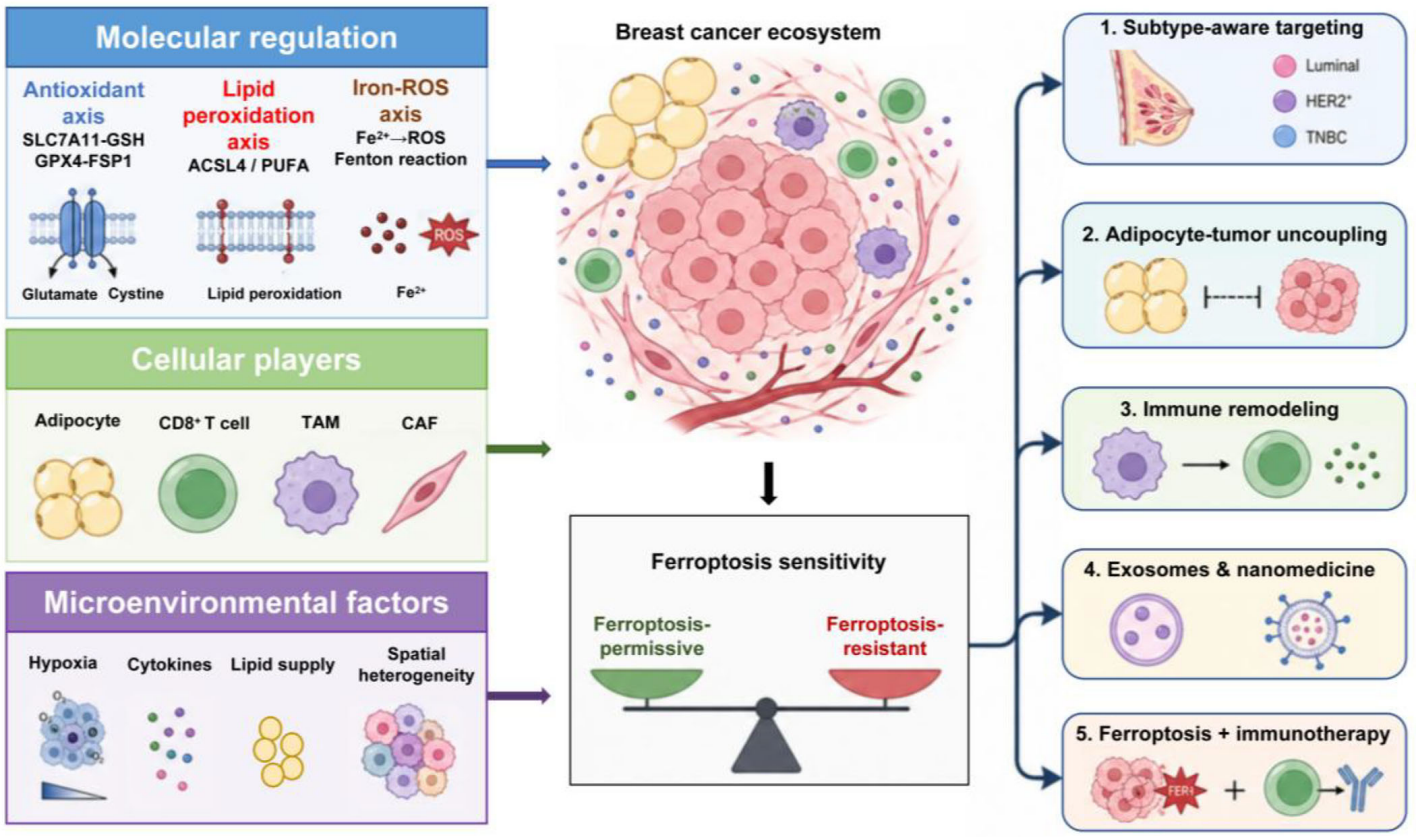
Roadmap of ferroptosis regulation and therapeutic integration in breast cancer. This schematic summarises the hierarchical organisation of ferroptosis regulation in breast cancer across molecular, cellular and microenvironmental levels. At the molecular level, ferroptosis is regulated by the balance between iron‐dependent reactive oxygen species (ROS) generation, lipid peroxidation pathways (e.g., ACSL4–PUFA axis) and antioxidant defence systems (e.g., SLC7A11–GSH–GPX4 and FSP1 pathways). At the cellular level, tumour cells interact dynamically with adipocytes, CD8^+^ T cells, tumour‐associated macrophages (TAMs) and stromal cells to modulate ferroptosis sensitivity. At the microenvironmental level, hypoxia, lipid availability, cytokine signalling and spatial metabolic heterogeneity further shape ferroptosis‐permissive versus ferroptosis‐resistant ecosystems. These regulatory layers collectively influence translational strategies, including subtype‐oriented targeting, immune checkpoint blockade (ICB), metabolic intervention, iron modulation and nanomedicine‐based therapy.

These regulatory layers have been increasingly incorporated into therapeutic design. Nanomaterial‐based systems enable tumour‐selective delivery of iron or redox‐active agents, thereby amplifying oxidative stress and inducing ferroptotic cell death. In parallel, photothermal, photodynamic and radiotherapeutic approaches can enhance lipid peroxidation and promote immunogenic cell death. Notably, immune checkpoint blockade (ICB) further reinforces ferroptosis through IFN‐γ‐mediated suppression of SLC7A11, establishing a functional link between antitumour immunity and lipid peroxidation. These interconnected regulatory layers and therapeutic opportunities are summarised in Figure 1. Such combinatorial strategies highlight ferroptosis as a convergence point between metabolic stress and immune activation.

Because the supporting evidence for these mechanisms varies across experimental systems, we introduce an evidence‐grading framework to distinguish breast‐cancer‐specific in vivo findings, preclinical models and extrapolations from other contexts. This framework, summarised in Table 2, is applied throughout the following sections to clarify the strength and translational relevance of each mechanism.

**TABLE 2:** Evidence grading of key mechanisms involved in the fat–immune–iron axis in breast cancer ferroptosis.

Evidence grade	Mechanistic theme	Representative finding
Strong^41^	CD8^+^ T cell–IFN‐γ promotes tumour ferroptosis.	IFN‐γ suppresses SLC7A11/SLC3A2 and enhances lipid peroxidation during immunotherapy.
Strong–moderate^29^, ^52^, ^82^	Adipocyte‐derived MUFA buffering suppresses ferroptosis.	MUFAs suppress ferroptosis by displacing peroxidation‐prone PUFAs in membranes.
Strong^81^	FADS1/2‐dependent PUFA remodelling shapes TNBC ferroptosis sensitivity.	PUFA remodelling directly alters ferroptosis vulnerability in TNBC.
Strong^83^, ^84^, ^94^	SCD1/FASN‐centred lipid desaturation drives ferroptosis resistance.	SCD1/FASN‐associated lipid protection raises ferroptosis threshold.
Moderate^59^, ^60^, ^61^, ^62^	TAM‐centred iron/redox buffering modulates ferroptosis threshold.	M1‐like macrophages favour pro‐oxidant stress; M2‐like macrophages buffer iron/lipid peroxidation.
Strong^47^, ^64^, ^65^	Tumour–macrophage crosstalk suppresses ferroptosis in TNBC.	Macrophage‐associated signalling supports progression/chemoresistance while restraining ferroptosis.
Moderate–strong^68^, ^69^, ^70^	Adipokine/AMPK‐connected signalling intersects with ferroptosis regulation.	AdipoR1–AMPK and related pathways alter multimodal death including ferroptosis.
Moderate–strong^75^	Hypoxia/acidosis create ferroptosis‐resistant niches.	Hypoxic and acidic microenvironments limit ferroptosis efficiency in TNBC.
Inferential^71^, ^72^, ^142^, ^143^	Spatially organised ferroptosis niches are plausible but not yet directly mapped.	Adipose‐border, immune–stromal and hypoxic zones likely differ in ferroptosis threshold.
Moderate–strong^22^, ^24^, ^90^, ^144^	Exosomes can either suppress or enhance ferroptosis.	Stromal exosomes may suppress ferroptosis, whereas engineered vesicles may sensitise ferroptosis and activate immunity.
Preclinical proof‐of‐concept^89^, ^91^, ^145^, ^146^	Nanomedicine can couple ferroptosis induction with immune remodelling.	Ferroptosis‐inducing nanoplatforms enhance immune activation or checkpoint responsiveness.
Strong^77^	Clinical translation remains early and biomarker‐limited.	Most evidence remains preclinical; biomarkers and toxicity control are unresolved.

## 3 ADIPOCYTES–TUMOUR METABOLIC COUPLING: LIPID AND REDOX SUPPLY IN FERROPTOSIS REGULATION

Adipocytes act as key stromal regulators of ferroptosis through extensive metabolic coupling with adjacent tumour cells. In breast cancer, the close anatomical proximity between malignant cells and mammary adipocytes creates a lipid‐rich microenvironment that directly influences ferroptotic sensitivity. One major mechanism involves the transfer of fatty acids. Cancer‐associated adipocytes (CAAs) undergo lipolysis during tumour progression, releasing free fatty acids (FFAs), glycerol and adipokines that are taken up by tumour cells for energy production and phospholipid synthesis.^27^, ^28^ This lipid influx reshapes membrane composition: PUFAs, incorporated via ACSL4 and LPCAT3, increase susceptibility to lipid peroxidation,^8^, ^9^ whereas monounsaturated fatty acids (MUFAs), particularly oleic acid, suppress peroxidation and confer resistance by stabilising membrane structure.^29^ In TNBC, adipocyte‐derived oleic acid has been shown to directly reduce ferroptotic vulnerability, illustrating that adipocyte–tumour coupling can determine ferroptosis sensitivity by modulating lipid composition rather than simply supplying nutrients.

In addition to lipid transfer, adipocytes regulate ferroptosis through redox metabolic support. Adipocyte‐derived metabolites, including lactate, pyruvate and glutamine, can replenish intracellular NADPH and GSH pools, thereby sustaining the GPX4–FSP1 antioxidant system.^30^, ^31^, ^32^, ^33^ This metabolic buffering reduces lipid peroxidation and raises the threshold for ferroptotic cell death. Adipokine signalling provides a further, albeit less well‐defined, regulatory layer. Leptin and adiponectin are known to influence pathways such as AMPK, JAK–STAT, PI3K–AKT and NRF2, which intersect with redox balance and lipid metabolism.^34^, ^35^, ^36^, ^37^, ^38^ However, direct evidence linking adipokines to ferroptosis regulation in breast cancer remains limited, and these effects are more appropriately interpreted as mechanistically suggestive rather than established drivers of ferroptosis resistance.

**FIGURE 2:**
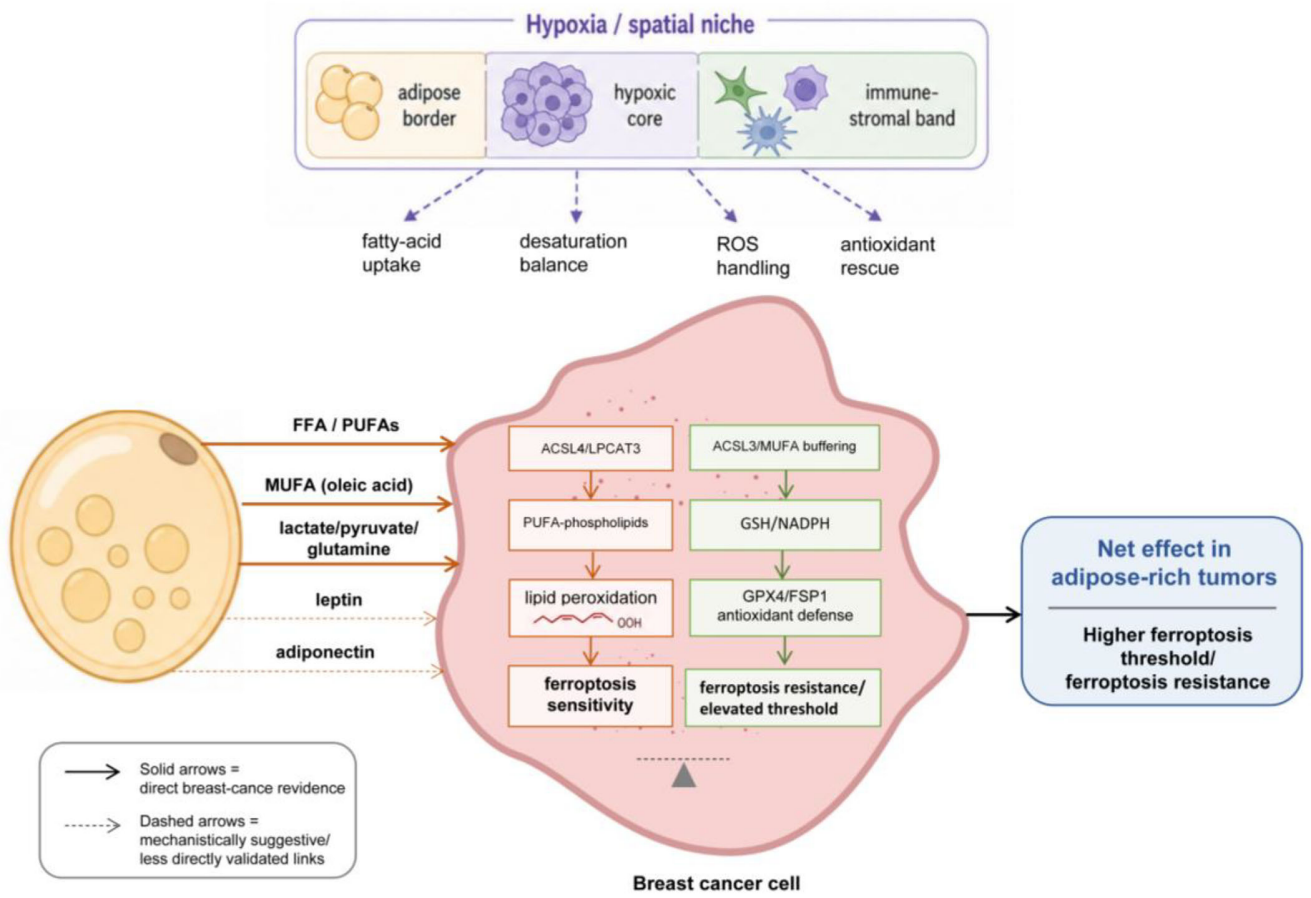
Adipocyte–tumour metabolic coupling and spatial regulation of ferroptosis in breast cancer. This figure illustrates how adipocyte‐derived lipid and redox metabolism regulates ferroptosis sensitivity in breast cancer cells. Polyunsaturated fatty acids (PUFAs) promote lipid peroxidation and ferroptosis susceptibility through ACSL4‐associated phospholipid remodelling, whereas monounsaturated fatty acids (MUFAs), particularly oleic acid, suppress membrane peroxidation and enhance ferroptosis resistance. Adipocyte‐derived metabolites including lactate, pyruvate, glutamine and glycerol support glutathione synthesis and antioxidant buffering. Adipokines such as leptin and adiponectin may further regulate ferroptosis through JAK–STAT, PI3K–AKT, AMPK and NRF2‐related pathways. Spatial heterogeneity, including hypoxic tumour cores and adipose‐border regions, dynamically alters lipid exchange, ROS handling and ferroptosis thresholds.

The impact of adipocyte–tumour coupling is also spatially and dynamically regulated within tumours. Spatial profiling studies indicate that regions adjacent to adipose tissue exhibit distinct lipid composition and antioxidant activity compared with central hypoxic areas.^39^ Hypoxia does not simply enhance oxidative stress but reprograms lipid uptake, desaturation and redox dependence, altering the balance between ferroptosis susceptibility and resistance.^40^ This spatial heterogeneity supports a model in which adipose‐border regions, hypoxic cores and immune‐infiltrated stromal zones impose distinct metabolic constraints on ferroptosis. Disrupting adipocyte‐derived lipid and redox support may therefore represent a viable strategy to sensitise breast tumours to ferroptosis‐based therapies. The major adipocyte‐derived lipid, redox and spatial regulatory mechanisms are summarised in Figure 2.

## 4 IMMUNE–TUMOUR CROSSTALK IN FERROPTOSIS REGULATION

The tumour immune microenvironment (TIME) shapes ferroptosis sensitivity through coordinated regulation of cytokine signalling, lipid metabolism and redox balance. Among immune populations, CD8^+^ cytotoxic T lymphocytes (CTLs) provide the most direct and well‐established pro‐ferroptotic pressure in breast cancer. Activated CTLs secrete IFN‐γ, which suppresses SLC7A11 and SLC3A2 expression, restricts cystine uptake and depletes intracellular GSH, thereby promoting lipid peroxidation and ferroptotic cell death.^41^ This pathway is further reinforced during ICB, linking ferroptosis induction to antitumour immunity.^41^, ^42^ In addition to redox regulation, IFN‐γ also promotes ACSL4‐dependent incorporation of PUFAs into phospholipids, increasing the availability of peroxidation‐prone substrates required for ferroptosis execution.^42^ These findings position CTLs as central drivers of ferroptosis through coordinated control of antioxidant capacity and lipid composition.

In contrast to CTLs, macrophage‐mediated regulation of ferroptosis is strongly context dependent. M1‐like macrophages are generally associated with a pro‐oxidant environment characterised by elevated ROS and reactive nitrogen species,^43^, ^44^, ^45^ whereas M2‐like macrophages exhibit antioxidant, iron‐retentive and tissue‐repair programs that buffer lipid peroxidation and promote ferroptosis resistance.^46^ Direct evidence from breast cancer now supports a dominant anti‐ferroptotic role for tumour‐associated macrophages (TAMs) in certain contexts. In TNBC, TAM‐derived TGF‐β1 activates the SMAD3–HLF axis in tumour cells, inducing GGT1 expression, preserving GSH/GSSG balance, sustaining GPX4 activity and suppressing ferroptosis while promoting tumour progression and cisplatin resistance.^47^ Tumour–macrophage communication further reinforces this phenotype: tumour‐derived exosomes enhance macrophage IDO1/KYN/NRF2‐dependent antioxidant adaptation, linking macrophage polarisation to ferroptosis resistance.^18^ These findings indicate that TAMs actively regulate cysteine metabolism, iron handling and redox buffering rather than merely modulating inflammation.

Ferroptosis within immune cells themselves produces divergent biological outcomes that depend on the affected cell type. In breast cancer, tumour‐infiltrating neutrophils can resist ferroptosis through Acod1–itaconate–NRF2 signalling, thereby maintaining persistence and promoting metastasis.^48^ Under certain conditions, ferroptotic neutrophils may instead release immunosuppressive mediators, including PGE2, IDO and oxidised lipids, which impair CD8^+^ T‐cell proliferation and cytotoxicity.^49^ These observations indicate that ferroptosis in the immune compartment is not uniformly beneficial and may, in specific contexts, reinforce immunosuppression.

**FIGURE 3:**
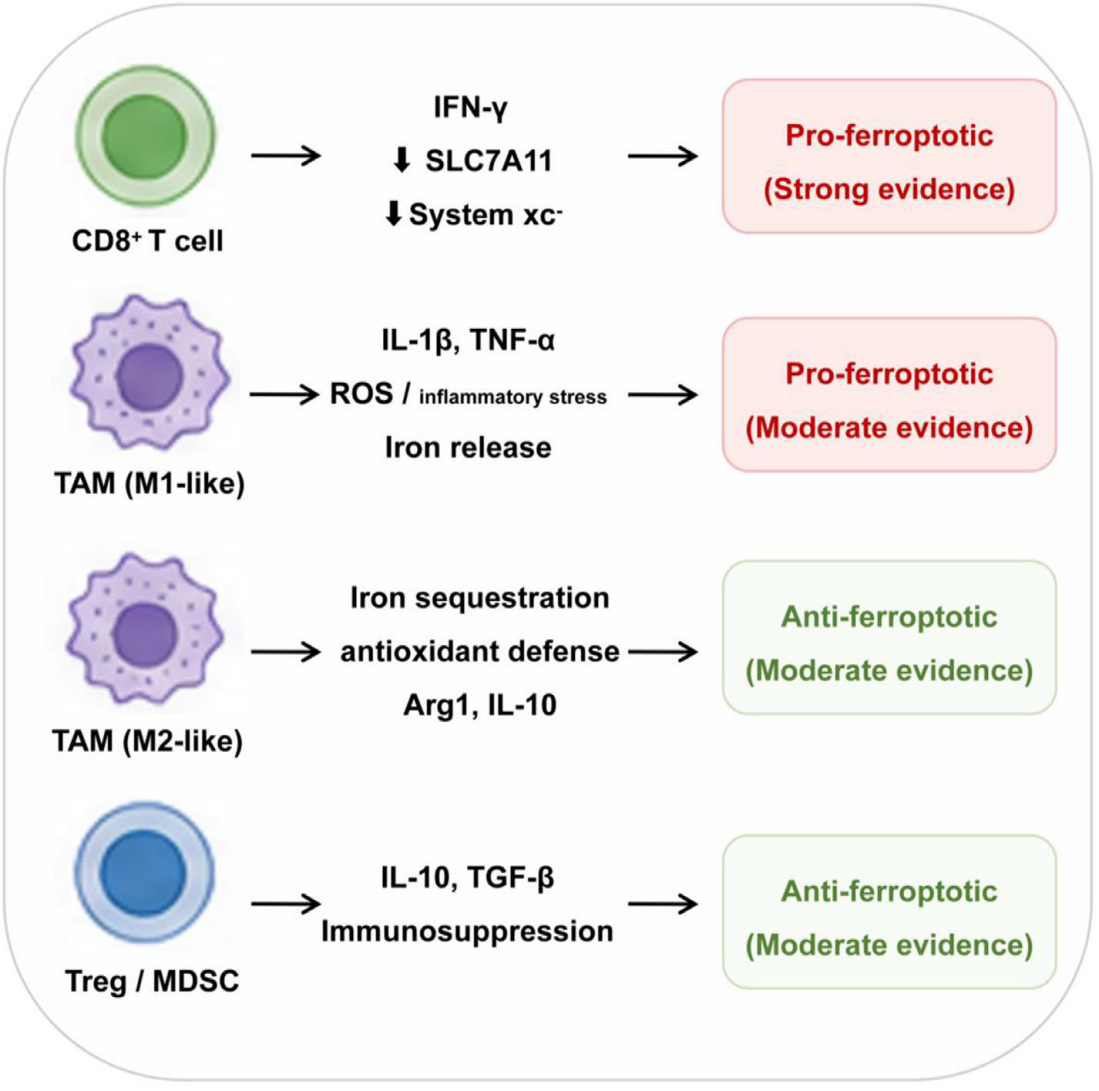
Immune–tumour crosstalk regulating ferroptosis in breast cancer. This schematic summarises the context‐dependent effects of immune cells on ferroptosis regulation in the breast tumour microenvironment. CD8^+^ T cells promote ferroptosis through interferon‐γ (IFN‐γ)‐mediated suppression of SLC7A11 and system xc^−^ activity, thereby limiting cystine uptake and glutathione synthesis. M1‐like tumour‐associated macrophages (TAMs) may enhance ferroptotic stress through inflammatory ROS production and iron release, whereas M2‐like TAMs contribute to ferroptosis resistance through iron buffering, antioxidant defence and immunosuppressive signalling. Regulatory T cells (Tregs) and myeloid‐derived suppressor cells (MDSCs) further suppress ferroptosis‐promoting immunity through IL‐10‐, TGF‐β‐ and redox‐associated immunosuppressive pathways. The overall outcome is highly context dependent and influenced by tumour subtype, metabolic state and immune composition.

Compared with CTLs and macrophages, ferroptosis regulation in Tregs and myeloid‐derived suppressor cells (MDSCs) remains less well defined. Emerging evidence suggests that intratumoural Tregs depend on lipid peroxide defence mechanisms to maintain their suppressive function,^50^ implying a role for ferroptosis‐related redox control in their stability. In contrast, MDSCs are primarily supported by indirect or state‐dependent evidence, with limited breast‐cancer‐specific validation.^51^ Taken together, the immune regulation of ferroptosis follows a clear evidence gradient: CTL‐mediated pro‐ferroptotic pressure is strongly established, macrophage and neutrophil‐mediated buffering now has direct support in breast cancer, Treg‐associated regulation is emerging and MDSC involvement remains largely inferential. This hierarchy underscores the need to consider immune context when designing ferroptosis‐based therapeutic strategies. Representative immune‐cell‐mediated ferroptosis regulatory pathways are summarised in Figure 3. Ferroptosis should therefore be interpreted as a context‐dependent process shaped by immune composition, lipid availability, redox state and spatial organisation within tumours. Key determinants of pro‐ versus anti‐ferroptotic outcomes are summarised in Table 3.

**TABLE 3:** Context‐dependent determinants of pro‐ versus anti‐ferroptotic outcomes in the breast tumour microenvironment.

Determinant	Pro‐ferroptotic context	Anti‐ferroptotic context	Main mechanistic explanation
Lipid species composition^29^, ^81^, ^82^	PUFA‐rich membrane state	MUFA‐rich membrane state	PUFAs favour lipid peroxidation; MUFAs buffer membrane susceptibility.
Lipid metabolic enzymes^8^, ^83^, ^84^, ^94^	ACSL4/LPCAT3/PUFA remodelling dominant	ACSL3/SCD1/FASN‐driven desaturation dominant	Different lipid‐rewiring programs alter ferroptosis readiness.
Macrophage polarisation^59^, ^60^, ^62^, ^64^	M1‐like/pro‐oxidant macrophage state	M2‐like/ferritin‐ and ferroportin‐buffering TAM state	Iron/redox handling differs between M1 and M2 programs.
CD8^+^ T‐cell / IFN‐γ signalling^41^, ^96^	Acute antitumour CTL pressure	Weak CTL pressure or immune‐cold ecosystem	IFN‐γ suppresses cystine transport and promotes ferroptotic pressure.
Immune‐cell ferroptosis itself^62^, ^147^	Preferential tumour‐cell ferroptosis with preserved antitumour immunity	Ferroptosis in beneficial immune cells or immunosuppressive buffering spared	Immune‐cell ferroptosis can either enhance or impair antitumour immunity depending on cell type/context.
Hypoxia/acidosis^75^	Limited/relieved hypoxia and acidosis	Persistent hypoxic and acidic stress	Hypoxia/acidosis can reduce ferroptosis efficiency and support adaptive buffering.
Spatial location within tumour^71^, ^72^, ^73^, ^143^	Iron‐loaded, PUFA‐rich, weakly buffered regions	Adipose‐border/immune‐buffered/hypoxic‐protected regions	Spatial niche organisation shapes local ferroptosis thresholds.
Adipocyte–tumour coupling^52^, ^66^, ^68^	Restricted lipid rescue/disrupted adipocyte support	Strong adipocyte‐derived MUFA and adipokine buffering	Adipocyte support modifies lipid composition and redox tone
Exosomal signalling^22^, ^24^, ^26^, ^90^	Engineered vesicles delivering ferroptosis‐sensitising cargo	Stromal/tumour exosomes delivering ferroptosis‐suppressive cargo	Exosomes can either inhibit or amplify ferroptosis and immune activation.

## 5 ADIPOSE–IMMUNE METABOLIC INTERPLAY: JOINT REGULATION OF FERROPTOSIS SENSITIVITY

**FIGURE 4:**
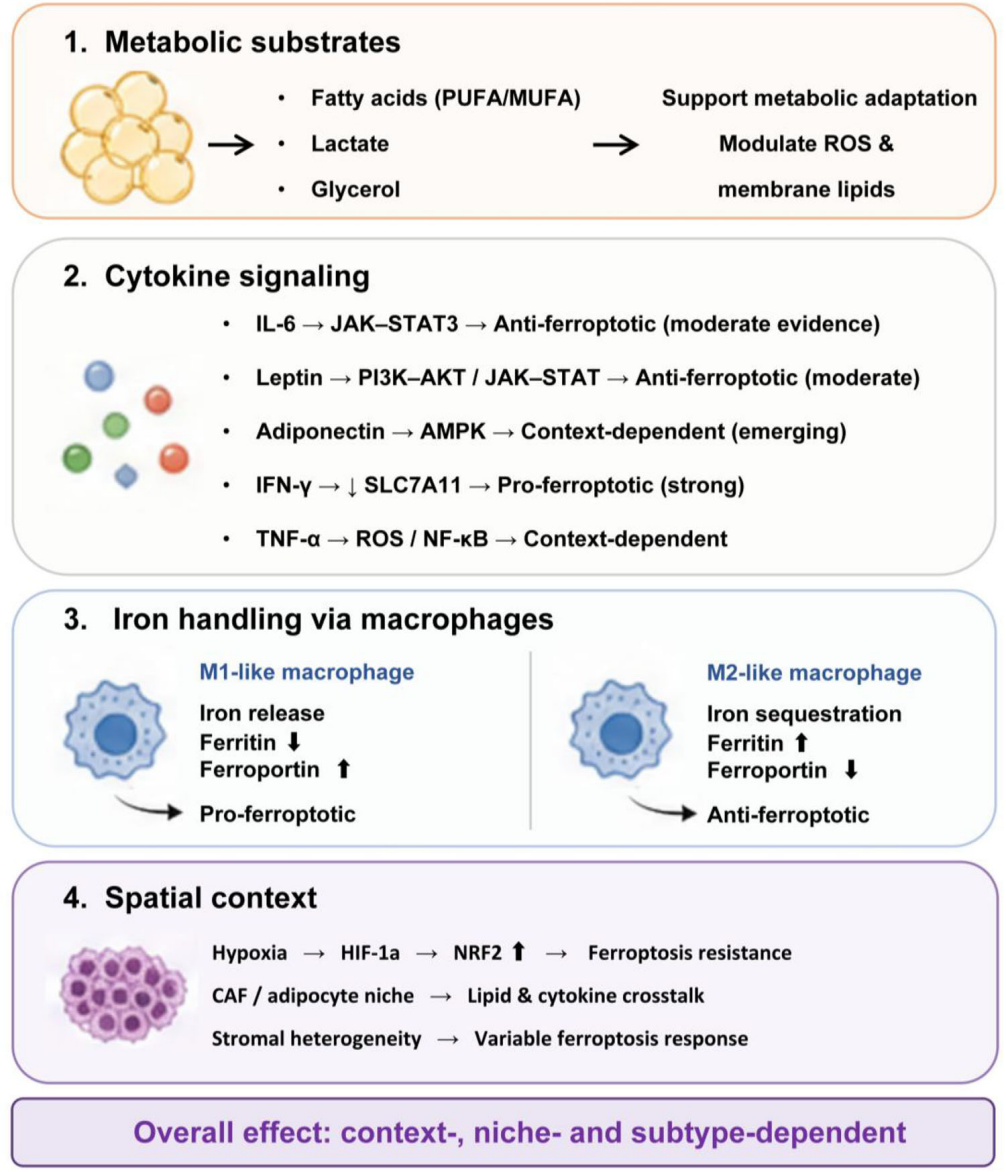
Adipose–immune metabolic interplay in ferroptosis regulation. This figure illustrates the integrated adipose–immune regulatory network controlling ferroptosis sensitivity in breast cancer. Adipocytes provide fatty acids, lactate and glycerol that modulate membrane composition, redox buffering and metabolic adaptation. Cytokines including IL‐6, leptin, adiponectin, IFN‐γ and TNF‐α regulate ferroptosis through JAK–STAT, PI3K–AKT, AMPK, NRF2 and inflammatory signalling pathways. Macrophage‐mediated iron handling further shapes ferroptotic stress through iron release, sequestration, ferritin buffering and antioxidant regulation. Spatial heterogeneity, particularly hypoxic and adipose‐rich niches, contributes to regional differences in ferroptosis sensitivity and metabolic adaptation within the same tumour ecosystem.

Rather than acting independently, adipocyte‐ and immune‐derived signals converge on a limited number of ferroptosis‐regulatory processes, including lipid availability, antioxidant buffering, iron distribution and spatial metabolic adaptation. The metabolic interplay between adipocytes and immune cells represents a critical but relatively underdefined layer of ferroptosis regulation in breast cancer. CAAs remodel the TME by releasing fatty acids, adipokines and inflammatory mediators that shape immune‐cell recruitment and function, including macrophages and regulatory T cells.^52^, ^53^, ^54^ Together with emerging evidence on ferroptosis–immunity crosstalk,^41^ these findings support a model in which adipose and immune components act in a coordinated manner rather than independently. Through combined control of metabolic substrate availability, iron distribution, cytokine signalling and spatial organisation, this adipose–immune axis modulates local ferroptosis sensitivity (Figure 4).

### 5.1 Metabolic coupling and energy exchange

CAAs undergo lipolysis and release FFAs, glycerol and lactate, which are utilised not only by tumour cells but also by immune populations such as TAMs and T cells.^27^, ^52^ In both adipose tissue and tumours, Tregs and macrophages preferentially rely on fatty‐acid uptake and fatty‐acid oxidation (FAO) to sustain their function.^55^ This FAO‐dependent program supports NADPH and GSH production, reinforcing antioxidant defences and limiting ROS accumulation.^56^ Although these studies do not directly assess ferroptosis, the same NADPH–GSH systems underpin GPX4‐mediated detoxification of lipid peroxides, suggesting that adipocyte‐derived fuels may indirectly elevate ferroptosis resistance by sustaining FAO‐dependent immunosuppressive cells.

Recent breast‐cancer evidence further indicates that metabolic competition within the TME can directly influence ferroptosis‐related responses. In TNBC, HEBP2‐governed glutamine competition between tumour cells and macrophages shapes immunotherapy efficacy,^57^ highlighting nutrient allocation as a component of ferroptosis‐relevant ecosystem regulation. At the same time, lipid effects are context dependent. While MUFA enrichment generally confers resistance, CD36‐mediated uptake can enhance sensitivity to palmitate‐induced ferroptosis in TNBC.^58^ These observations indicate that the ferroptotic consequence of adipose‐derived lipid exposure depends on lipid species, uptake pathways and the surrounding immune–redox context.

### 5.2 Iron pool and macrophage polarisation

Adipose‐associated macrophages play a central role in regulating ferroptosis through iron handling and redox control. M1‐like macrophages accumulate labile iron and generate high levels of ROS/RNS, creating a pro‐oxidant environment that favours lipid peroxidation.^59^ In contrast, M2‐like macrophages preferentially sequester iron in ferritin and export it via ferroportin, thereby establishing an iron‐buffered, antioxidant state.^59^, ^60^ Bidirectional interactions further link ferroptosis and macrophage function. Tumour‐cell ferroptosis can promote M2 polarisation through uptake of KRAS‐containing vesicles released from ferroptotic cells,^61^ reinforcing an immunosuppressive environment. Conversely, macrophages themselves display differential ferroptosis sensitivity, with iron‐loaded M1 macrophages being more susceptible, whereas selective targeting of M2 TAMs may relieve immunosuppression.^62^

In breast cancer, direct evidence supports a dominant anti‐ferroptotic role for TAMs in specific contexts. Tumour–macrophage crosstalk suppresses ferroptosis through TGF‐β1–SMAD3–HLF–GGT1 signalling,^47^ while M2‐like TAMs dynamically restrain ferroptotic responses in TNBC.^63^ Additional studies link ferroptosis‐related metabolic programs to macrophage polarisation and immune remodelling,^64^ including the USP8/CEP55/CHMP6 axis, which coordinates ferroptosis regulation with M2 polarisation.^65^ These findings position macrophage polarisation as a key determinant of iron–redox balance and ferroptosis sensitivity in the TME.

### 5.3 Cytokine bridging and immunometabolic rewiring

Adipocyte‐derived cytokines and adipokines provide an additional regulatory layer linking metabolism to immune function. IL‐6–JAK/STAT3 signalling promotes mitochondrial metabolism and supports macrophage polarisation towards immunosuppressive states.^53^ Leptin enhances macrophage activation and T‐cell metabolic fitness through JAK2–STAT3 and PI3K–AKT pathways,^66^ whereas adiponectin activates AMPK and is associated with anti‐inflammatory and redox‐regulatory effects.^37^, ^38^ Immune‐derived cytokines further modulate ferroptosis sensitivity. Pro‐inflammatory mediators such as TNF‐α and IL‐1β increase oxidative stress and lipid peroxidation, whereas IL‐4 and IL‐13 promote M2 polarisation and activate NRF2/GPX4‐dependent antioxidant programs.^59^, ^67^

More direct links between adipokine‐related signalling and ferroptosis are now emerging in breast cancer. The AdipoR1–AMPK axis suppresses tumour growth through multimodal cell death pathways, including ferroptosis.^68^ Consistently, AMPK activation has been shown to promote ferroptosis in TNBC and tamoxifen‐resistant breast cancer via Foxo3‐ and BECN1–SLC7A11‐dependent mechanisms.^69^, ^70^ These findings support a model in which adipose–immune interactions function as a cytokine–metabolic bridge that determines whether local tumour regions adopt ferroptosis‐permissive or ferroptosis‐buffered states.

### 5.4 Spatial heterogeneity and ferroptosis niches

Spatial organisation further constrains ferroptosis regulation in breast cancer. Multiplexed imaging and spatial transcriptomic studies have revealed recurrent tissue architectures, including T cell‐enriched invasive margins, macrophage‐dominated stromal regions and immune‐excluded tumour cores.^71^, ^72^ These regions differ in lipid metabolism, oxidative stress responses and immune signalling and are associated with distinct clinical outcomes.^73^ Peritumoural adipose tissue represents a specialised niche in which CAAs and immune cells jointly regulate lipid availability and redox balance.^74^ Functional studies indicate that hypoxic and acidic microenvironments can suppress ferroptosis in TNBC,^75^ suggesting that spatially separated tumour regions may exhibit distinct ferroptosis thresholds despite shared genetic backgrounds.

Although ferroptosis has not yet been directly mapped at single‐cell spatial resolution, current evidence supports a model of spatially organised ferroptosis niches. Adipocyte‐ and immune‐rich border regions, characterised by FAO activity, NADPH/GSH metabolism and iron‐buffering macrophages, are likely to be relatively ferroptosis‐resistant. In contrast, PUFA‐rich, iron‐loaded tumour regions distant from adipose and immune support may be more ferroptosis‐prone. Validation of this model will require integrated spatial transcriptomic and metabolomic analyses incorporating ferroptosis‐specific markers such as ACSL4, GPX4/FSP1 and iron‐storage pathways.^39^, ^41^

## 6 TRANSLATIONAL OPPORTUNITIES: ECO‐FERROTHERAPY – REWIRING THE METABOLIC ECOSYSTEM TO SENSITISE FERROPTOSIS

**FIGURE 5:**
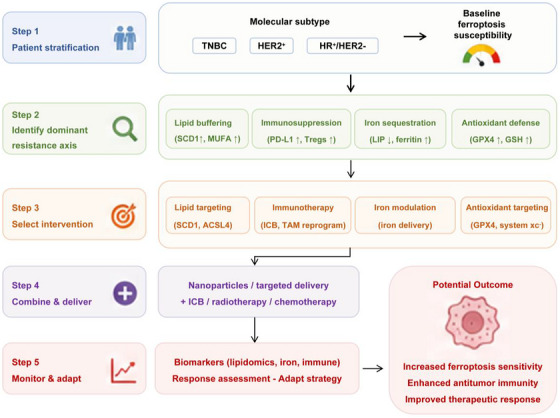
Eco‐ferrotherapy framework for precision combination strategies in breast cancer. This decision‐tree framework summarises ecosystem‐oriented ferroptosis‐targeting strategies in breast cancer. The first layer stratifies tumours according to molecular subtype and baseline ferroptosis ecosystem status. The second layer identifies dominant resistance mechanisms, including lipid buffering, immunosuppression, iron sequestration and antioxidant defence. The third layer proposes corresponding therapeutic interventions, including lipid‐targeting strategies, immunotherapy, iron amplification approaches and GPX4/system xc^−^ inhibition. Combination modalities involving nanomedicine, immune checkpoint blockade (ICB), radiotherapy and chemotherapy are integrated to enhance ferroptosis sensitivity and overcome stromal resistance. The final goal is to improve therapeutic response through coordinated ecosystem‐level modulation rather than isolated ferroptosis induction.

Ferroptosis sensitivity in solid tumours is increasingly recognised as an emergent property shaped by the broader tumour ecosystem rather than a tumour‐intrinsic feature alone. Stromal and immune components – including adipocytes, macrophages and vascular niches – collectively regulate lipid availability, redox balance and iron distribution, thereby determining ferroptotic responsiveness.^76^, ^77^ Within this context, we define eco‐ferrotherapy as a translational framework that integrates tumour‐intrinsic ferroptosis induction with disruption of extrinsic buffering systems. A ferroptosis‐permissive ecosystem is therefore characterised by (i) a lipid/iron state favouring peroxidation, (ii) reduced stromal antioxidant buffering and (iii) preserved antitumour immune pressure, particularly CD8^+^ T‐cell activity.^78^ This framework prioritises coordinated intervention across tumour, stromal and immune compartments rather than single‐agent ferroptosis induction (Figure 5).

### 6.1 Disrupting adipocyte‐derived lipid buffering

Adipocytes‐derived MUFAs represent a major source of ferroptosis resistance by stabilising membrane lipids and suppressing peroxidation. Exogenous MUFAs inhibit ferroptosis by displacing peroxidation‐prone PUFAs, while SCD1‐driven desaturation establishes a lipid environment resistant to oxidative damage.^29^, ^79^, ^80^ Targeting this pathway provides a direct strategy to sensitise tumours: pharmacological SCD1 inhibition or restriction of MUFA availability shifts membrane composition towards PUFA enrichment, enhancing the efficacy of GPX4 inhibition and system xc^−^ blockade.^80^ Breast‐cancer‐specific studies further support this approach. FADS1/2‐mediated PUFA remodelling regulates ferroptosis susceptibility in TNBC, while tumour‐derived MUFAs and selenoprotein‐dependent pathways contribute to ferroptosis resistance and metastatic progression.^81^, ^82^ In parallel, SCD1‐centred regulatory circuits – including CircSCD1 stabilisation and mTORC1/SREBP1/SCD1 signalling – function as key ferroptosis defence modules, and their disruption restores ferroptotic sensitivity.^83^, ^84^, ^85^ These findings establish lipid‐buffering blockade as a practical entry point for eco‐ferrotherapy.

### 6.2 Reprogramming TAM‐mediated iron and redox buffering

TAMs are central regulators of iron availability and redox balance within the TME. M1‐like macrophages promote oxidative stress through labile iron accumulation and ROS/RNS production, whereas M2‐like macrophages sequester and export iron, generating an antioxidant niche that protects tumour cells.^46^, ^53^ This polarisation‐dependent iron handling directly influences ferroptosis susceptibility.

Therapeutically, reprogramming TAMs or reducing their immunosuppressive activity can relieve ferroptosis resistance. Inhibition of CSF1R or CCR2 signalling reduces M2 macrophage abundance and shifts the balance towards pro‐oxidant states.^86^ Breast‐cancer studies further demonstrate that tumour–macrophage crosstalk actively suppresses ferroptosis and promotes therapy resistance, while ferroptosis‐related metabolic programs are tightly linked to M2 polarisation and immune remodelling.^47^, ^63^, ^64^ These findings support combined strategies integrating ferroptosis induction with TAM‐directed interventions to amplify ferroptotic pressure.

### 6.3 Integrating ferroptosis with immunotherapy

Ferroptosis intersects with adaptive immunity through well‐defined molecular mechanisms. IFN‐γ released by CD8^+^ T cells suppress SLC7A11/SLC3A2 expression, limits cystine uptake and promotes lipid peroxidation during ICB.^41^ This establishes ferroptosis as both a downstream effector and an amplifier of antitumour immunity. Ferroptosis can also enhance tumour immunogenicity by releasing oxidised lipids and damage‐associated signals that promote dendritic‐cell activation and T‐cell priming.^59^ Accordingly, combining ferroptosis inducers with immune‐checkpoint inhibitors represents a rational therapeutic strategy. Preclinical data suggest that sequential strategies – ferroptosis priming followed by immunotherapy – may further enhance efficacy, although optimal timing remains to be defined.

### 6.4 Engineering ferroptosis using nanomedicine

Nanotechnology enables spatially targeted modulation of ferroptosis and the TME. Ferroptosis‐based nanoplatforms – including iron‐delivery systems, GPX4 inhibitors and catalytic nanomaterials – can increase labile iron and lipid peroxidation within tumours.^87^ Importantly, these systems can be engineered to combine ferroptosis induction with immune modulation or redox‐responsive drug release. In breast cancer, emerging nanoplatforms integrate ferroptosis sensitisation with immune remodelling, improving drug delivery and overcoming stromal buffering.^87^, ^88^, ^89^ Beyond delivery, such platforms may actively reprogram tumour ecosystems by simultaneously enhancing oxidative stress, disrupting antioxidant defences and modulating immune‐cell function. This positions nanomedicine as a key tool for ecosystem‐level ferroptosis control.

### 6.5 Harnessing exosome‐mediated modulation

Exosome‐based approaches provide a complementary strategy for coupling ferroptosis induction with microenvironmental modulation. Endogenous tumour‐ or stromal‐derived exosomes can suppress ferroptosis – for example, CAF‐derived vesicles inhibit ACSL4 and lipid peroxidation – whereas engineered vesicles can deliver ferroptosis‐sensitising cargo and enhance immune activation.^24^, ^26^, ^90^ Recent breast‐cancer studies demonstrate that vesicle‐based platforms can combine ferroptosis induction with immunomodulation, enhancing dendritic‐cell maturation and T‐cell infiltration.^89^, ^91^ In this context, ferroptosis functions not only as a cytotoxic mechanism but also as an immunogenic amplifier. These approaches extend eco‐ferrotherapy beyond tumour‐cell targeting to include modulation of stromal communication and metastatic niche formation.^92^

### 6.6 Towards a clinical ferroptosis ecosystem score

**FIGURE 6:**
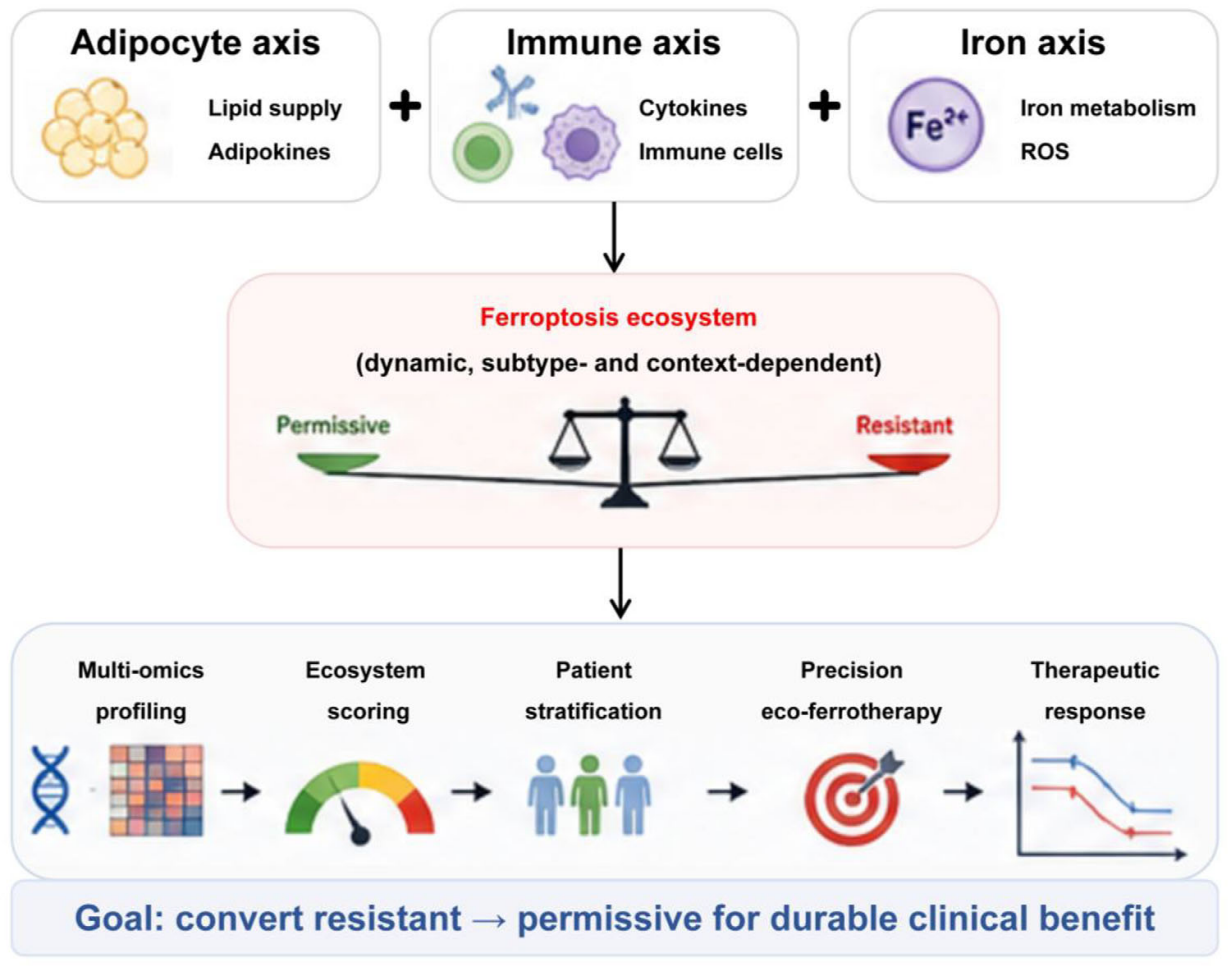
Integrative ferroptosis ecosystem framework and translational pipeline in breast cancer. This figure presents an integrated conceptual framework linking adipocyte‐derived metabolic regulation, immune‐mediated ferroptosis modulation and iron‐dependent oxidative stress into a unified ferroptosis ecosystem model. These interconnected axes collectively determine whether the tumour microenvironment remains ferroptosis‐resistant or becomes ferroptosis‐permissive. The lower panel illustrates a proposed translational pipeline integrating multi‐omics profiling, ecosystem scoring, biomarker‐guided patient stratification and precision eco‐ferrotherapy strategies. This framework highlights the potential clinical application of ecosystem‐oriented ferroptosis modulation for improving personalised breast cancer therapy.

Translation of eco‐ferrotherapy will require biomarker systems that capture ecosystem‐level ferroptosis sensitivity. Existing transcriptomic indices linking lipid metabolism, ferroptosis and immune infiltration provide initial frameworks but remain retrospective and not specifically predictive of ferroptosis‐directed therapies.^16^, ^93^ A clinically relevant scoring strategy should integrate three core dimensions: tumour‐intrinsic ferroptosis readiness (e.g., ACSL4/GPX4 balance and lipid‐protection pathways), immune pressure (e.g., CD8^+^ T‐cell activity and IFN‐γ signalling) and microenvironmental buffering (e.g., myeloid iron handling and adipocyte‐derived lipid support).^16^, ^94^, ^95^ Notably, subtype‐specific differences in ferroptosis vulnerability should be considered before applying such a framework (Table 4). Candidate markers and scoring axes are summarised in Table 5. An integrated ecosystem‐oriented framework linking adipocyte, immune and iron axes to clinical translation is summarised in Figure 6. At present, this score should be viewed as a conceptual and testable model requiring prospective validation.

**TABLE 4:** Subtype‐oriented translational roadmap for eco‐ferrotherapy in breast cancer.

Breast cancer subtype	Key ferroptosis‐related features	Most relevant therapeutic direction	Evidence level	Main limitation
TNBC (heterogeneous)^13^, ^41^	Marked ferroptosis heterogeneity; immune‐linked regulation	Combine ferroptosis induction with immunotherapy (e.g., ICB)	Direct TNBC mechanistic/preclinical	Heterogeneity; biomarker selection
LAR‐TNBC^13^, ^148^	AR/GPX4–GSH‐dependent ferroptosis resistance	Target GPX4 or glutathione metabolism	Direct subtype‐specific TNBC evidence	LAR subgroup selection required
Basal/QNBC TNBC^149^	ACSL4‐driven lipid peroxidation sensitivity	Lipid metabolism‐based ferroptosis targeting	Direct breast‐cancer subtype evidence	Limited clinical validation
Adaptive/drug‐tolerant TNBC^150^	GPX4 suppression with compensatory FSP1 upregulation	Dual GPX4–FSP1 targeting	Direct TNBC mechanistic evidence	Adaptive resistance; toxicity concerns
ER^+^/HR^+^ ^151^	Estrogen‐regulated system xc^−^ and redox buffering	Combine ferroptosis sensitisation with endocrine therapy	Direct ER^+^ breast‐cancer evidence	Mostly preclinical evidence
Tamoxifen‐resistant ER^+^ ^152^	Ferroptosis escape contributes to drug resistance	Ferroptosis‐based re‐sensitisation strategies	Direct ER^+^ tamoxifen‐resistance evidence	Early‐stage evidence (limited validation)
Luminal subtype^153^	Cystine transport supports antioxidant defence	Target cystine uptake/redox metabolism	Direct luminal breast‐cancer molecular evidence, ferroptosis‐relevant	Indirect ferroptosis evidence
ER^+^/HER2^+^ resistant^154^	Therapy‐induced metabolic vulnerabilities	Exploratory ferroptosis‐based combinations	Subtype‐relevant but not ferroptosis‐specific primary evidence	Not ferroptosis‐specific primary evidence
HER2^+^ ^155^	Emerging ferroptosis‐targeted nanotherapy approaches	HER2‐directed ferroptosis delivery systems	Direct HER2^+^ preclinical nanotherapy evidence	Preclinical stage; delivery challenges
Pan‐subtype^156^	Ferroptosis sensitivity varies across tumours	Biomarker‐guided patient stratification (e.g., FERscore)	Multi‐cohort/computational + validation evidence	Lack of prospective clinical validation

**TABLE 5:** Proposed framework for a clinical ferroptosis ecosystem score in breast cancer.

Score axis	Candidate markers/readouts	Biological meaning	Evidence status
Tumour‐intrinsic ferroptosis readiness^13^, ^156^	FERscore; ACSL4/GPX4‐related markers	Baseline ferroptosis‐inducer sensitivity	Breast cancer cohort/model validation
Lipid‐protection axis^82^, ^83^, ^94^	SCD1, FASN, MUFA‐related programs	Lipid desaturation and anti‐ferroptotic buffering	Breast cancer mechanistic evidence
Cystine/redox‐buffering axis^151^, ^153^	SLC7A11/system xc^−^, cystine transporter programs	Cystine uptake, GSH synthesis, endocrine‐linked redox defence	ER^+^/luminal breast cancer evidence
Immune–pressure axis^41^	CD8^+^ T‐cell infiltration; IFN‐γ signature	Endogenous pro‐ferroptotic immune pressure	Strong mechanistic/immunotherapy‐linked evidence
Immunotherapy‐resistance axis^95^	PSAT1–GPX4 adaptation	Ferroptosis escape associated with reduced immunotherapy efficacy	Emerging breast cancer translational evidence
Myeloid buffering axis^47^, ^48^	TAM/M2 markers; neutrophil Acod1–itaconate–Nrf2 axis	Immune‐cell redox buffering and ferroptosis resistance	Breast cancer mechanistic evidence
Adipocyte/stromal buffering axis^52^, ^66^	Adipocyte‐rich niche; MUFA supply; leptin/adiponectin context	Extrinsic lipid rescue and stromal redox support	Breast cancer mechanistic + supportive adipokine evidence
Spatial validation layer^39^, ^73^, ^143^	Spatial transcriptomics/proteomics plus ferroptosis markers	Regional ferroptosis‐prone vs. ferroptosis‐buffered niches	Breast cancer spatial atlas/inferential

### 6.7 Clinical status and translational barriers

Clinical translation of ferroptosis‐based therapy in breast cancer remains at an early stage. Most evidence derives from mechanistic studies and preclinical models rather than clinical trials using dedicated ferroptosis inducers. Current priorities therefore focus on biomarker‐guided combination strategies, selective delivery systems and approaches that preserve antitumour immunity while inducing tumour ferroptosis.^96^ Key barriers include off‐target toxicity, pharmacokinetic limitations, adaptive antioxidant resistance and the absence of validated predictive biomarkers. From a clinical perspective, TNBC – particularly immune‐inflamed or stromal‐rich subtypes – represents the most actionable context, whereas HR^+^ and HER2^+^ disease may require biomarker‐driven strategies rather than empirical ferroptosis induction. Subtype‐specific therapeutic opportunities and limitations are summarised in Table 4.

### 6.8 Limitations of current knowledge and future priorities

Despite rapid progress, several challenges remain. Most studies examine isolated components of the tumour ecosystem, and direct in vivo evidence for integrated fat–immune–iron regulation is limited. Distinguishing adipocyte‐derived buffering from immune‐cell‐mediated effects also remains difficult in adipose‐rich tumours. In addition, no ferroptosis‐targeted therapies are currently approved for breast cancer, and more physiologically relevant models are needed. Future priorities include developing predictive biomarkers, defining optimal combinations with immunotherapy and avoiding unintended ferroptosis in immunologically beneficial cell populations. Addressing these challenges will be essential for translating eco‐ferrotherapy into clinically effective strategies.

## AUTHOR CONTRIBUTIONS

**Juan Sun**: Conceptualization, Literature search, Writing—original draft, Writing—review and editing. **Yang Qu**: Literature search, Investigation. **Ru Yao**: Literature screening, Investigation. **Yidong Zhou**: Supervision, Conceptualization, Writing—review and editing, Project administration. Juan Sun, Yang Qu, and Ru Yao are co‐first authors. Yidong Zhou is the corresponding author. All authors have read and approved the final manuscript.

## CONFLICT OF INTEREST STATEMENT

The authors declare no conflicts of interest.

## Data Availability

The datasets are available from the corresponding author.
